# Conserved mutation of Epstein-Barr virus-encoded BamHI-A Rightward Frame-1 (BARF1) gene in Indonesian nasopharyngeal carcinoma

**DOI:** 10.1186/1750-9378-5-16

**Published:** 2010-09-19

**Authors:** Susanna H Hutajulu, Eveline K Hoebe, Sandra AWM Verkuijlen, Jajah Fachiroh, Bambang Hariwijanto, Sofia M Haryana, Servi JC Stevens, Astrid E Greijer, Jaap M Middeldorp

**Affiliations:** 1 Faculty of Medicine/Dr. Sardjito Hospital, Universitas Gadjah Mada, Yogyakarta, Indonesia; 2 Department of Pathology, VU University Medical Center, Amsterdam, The Netherlands; 3 Department of Clinical Genetics, Maastricht University Medical Center, Maastricht, The Netherlands

## Abstract

**Background:**

BamHI-A rightward frame-1 (BARF1) is a carcinoma-specific Epstein-Barr virus (EBV) encoded oncogene. Here we describe the BARF1 sequence diversity in nasopharyngeal carcinoma (NPC), other EBV-related diseases and Indonesian healthy EBV carriers in relation to EBV genotype, viral load and serology markers. Nasopharyngeal brushings from 56 NPC cases, blood or tissue from 15 other EBV-related disorders, spontaneous B cell lines (LCL) from 5 Indonesian healthy individuals and several prototype EBV isolates were analysed by PCR-direct sequencing.

**Results:**

Most NPC isolates revealed specific BARF1 nucleotide changes compared to prototype B95-8 virus. At the protein level these mutations resulted in 3 main substitutions (V29A, W72G, H130R), which are not considered to cause gross tertiary structure alterations in the hexameric BARF1 protein. At least one amino acid conversion was detected in 80.3% of NPC samples compared to 33.3% of non-NPC samples (p < 0.001) and 40.0% of healthy LCLs (p = 0.074). NPC isolates also showed more frequent codon mutation than non-NPC samples. EBV strain typing revealed most isolates as EBV type 1. The viral load of either NPC or non-NPC samples was high, but only in non- NPC group it related to a particular BARF1 variant. Serology on NPC sera using IgA/EBNA-1 ELISA, IgA/VCA-p18 ELISA and immunoblot score showed no relation with BARF1 sequence diversity (p = 0.802, 0.382 and 0.058, respectively). NPC patients had variable antibody reactivity against purified hexameric NPC-derived BARF1 irrespective of the endogenous BARF1 sequence.

**Conclusion:**

The sequence variation of BARF1 observed in Indonesian NPC patients and controls may reflect a natural selection of EBV strains unlikely to be predisposing to carcinogenesis. The conserved nature of BARF1 may reflect an important role in EBV (epithelial) persistence.

## Background

Epstein-Barr virus (EBV) is a human gamma-herpes virus that establishes persistent infection of B lymphocytes in more than 90% of the human adult population [1,2]. EBV is associated with a variety of lymphocytic cell malignancies including Burkitt lymphoma, Hodgkin's Disease, non-Hodgkin lymphoma, and post-transplant and AIDS-associated lymphoproliferative disorders as well as epithelial cell malignancies such as nasopharyngeal carcinoma (NPC) and gastric carcinoma, reflecting its dual cell tropism [3].

The vast majority of NPC cases, in particular the prevalent undifferentiated WHO type-III, is 100% related to EBV infection. In this type of NPC, EBV is present in all tumour cells with active expression of distinct viral coding and non coding genes [4]. The etiological role of EBV in NPC pathogenesis is consistently reflected by the presence of clonal EBV genomes in premalignant and malignant nasopharyngeal lesions [5] and the expression of viral proteins [4]. NPC develops in parallel with characteristic IgA and IgG antibody responses to EBV proteins which are detectable at early and even preclinical stage of disease [6-8]. Moreover, abnormal EBV DNA loads and viral mRNA are present in nasopharyngeal brushings, whereas elevated EBV DNA loads are detectable in the circulation of most patients at the time of NPC diagnosis [7,9-11].

Two types of EBV have been identified, which can be discriminated by genomic difference in a subset of latent genes encoding EBV nuclear antigen 2 (EBNA-2), EBNA-3A, -3B, and -3C [12,13]. EBV type 1, which is predominantly detected in Asian NPC patients, has a greater efficiency of B lymphocyte transformation than EBV type 2 [14], and this difference may associate with divergence in the EBNA-2 sequences [15,16].

EBV gene expression between and within tumors is highly variable. Each EBV-associated malignancy is characterized by a distinct viral gene expression profile, although EBNA-1, EBERs, and BARTs are found in all EBV-related tumors [4]. Of the 86 genes encoded by EBV genome [17], two are considered as viral oncogenes, i.e. LMP1 and BARF1, both having tumorigenic activity in an epithelial background [2,18-20].

LMP1 is detected only in 50-90% of NPC tumors with heterogenous expression [21-23]. LMP1 gene with a 30 bp deletion (del-LMP1) at nucleotide positions 168256-168285 (codons 346-355) compared to the B95-8 EBV prototype is found in a higher frequency of Asian NPC cases [24,25]. In addition, EBV del-LMP 1 is associated with increased NPC tumorigenicity in nude mice [26]. More recently, additional amino acid substitutions have been described for LMP1, but the functional consequences remain unclear [27,28].

BARF1-encoded protein was found to be expressed in more than 85% NPC biopsies and its mRNA is consistently expressed [10,29-31]. BARF1 protein forms a hexameric structure that is actively secreted and may act as immunomodulator through homology with the colony stimulating factor receptor c-fms, binding CSF-1 (M-CSF) [17,30-35]. BARF1 has mitogenic properties and can induce oncogenic transformation in rodent fibroblasts [18,19,35] and human B lymphocytic line [36,37]. Overexpression of BARF1 in EBV-infected epithelial cells leads to enhanced tumor formation in nude mice [20] and its immortalization potency has been demonstrated in monkey kidney primary epithelial cells [38]. Moreover, the BARF1 gene is consistently transcribed in EBV-positive gastric cancer in the absence of LMP1 [31,39]. Thus, BARF1 would then be more pivotal for NPC oncogenesis than the LMP1 protein. However, in contrast to LMP1 which has been widely explored, study of BARF1 sequence variation is still limited.

Therefore in the present study we aimed to determine whether BARF1 gene has sequence diversity in NPC. To verify its association with NPC, sequence analysis in other EBV-related disorders and in spontaneous lymphoblastoid cell lines (LCL) established from healthy Indonesian blood donors was also defined. To identify whether BARF1 variation has an influence on tumor biology, EBV genotype (EBNA-2 subtype), EBV DNA load, and serology pattern were analysed. Finally, we studied the immunogenic properties of NPC-derived BARF1 as a purified hexameric protein.

## Results

### Most of NPC isolates display nucleotide and amino acid changes

In the present study we have sequenced the complete BARF1 gene from 56 NPC brushing samples,15 non-NPC EBV-related tissue or blood, 5 spontaneous LCL from healthy EBV-seropositive individuals and several NPC-derived and non-NPC reference EBV strains. The EBV genome of B95-8 was used as the prototype EBV strain. The sequence of BARF1 gene derived from the B95-8 transformed LCL JY was identical to that of the B95-8 sequence from the database (GeneBank No. V01555). Prototype GD1 and AG876 strains also revealed an identical BARF1 sequence compared to B95-8. The C666-1 cell line showed 1 silent mutation at position of 165545 (T to C). The C15 tumor line displayed 4 nucleotide mutations, one at position of 165589 (T to C) leading to V29A conversion, and 3 silent mutations at position of 165677 (G to A), 165944 (C to T), and 166136 (C to T). The C17 tumor line did not demonstrate any nucleotide exchange compared to the B95-8 prototype.

Fourty-five (80.3%) NPC isolates, 5 (33.3%) non-NPC EBV isolates, and 2 (40.0%) healthy LCLs exhibited nucleotide mutations relative to the B95-8 prototype sequence (table 1). A total of 11 different nucleotide mutations were identified, scattered over the BARF1 sequence. Most NPC isolates showed multiple point mutations within the BARF1 nucleotide sequence. At the protein level most NPC (78.6%) demonstrated 2 amino acid substitutions at position V29A (valine to alanine) and H130R (histidine to arginine), except 1 sample (NPC-13) which displayed a unique conversion in the position of W72G and H130R. Some isolates (14.3%) showed rare mutations at position W72G (tryptophan to glycine). Very rare mutations at position S12T (serine to threonine) and S128F (serine to phenylalanine) were also observed in 2 single NPC samples, together with the above-mentioned more prevalent V29A and H130R changes. Other nucleotide mutations did not result in amino acid substitutions and were therefore silent.

**Table 1:** The point nucleotide mutations and amino acid changes observed in the BARF1 gene sequence from 56 NPC patients, 15 other EBV-related disorders, and 5 healthy individuals (one isolate might show more than one mutation).

**Nucleotide no**.	Nucleotide change	**AA No**.	AA change	NPC (n = 56)	Non-NPC (n = 15)	Healthy (n = 5)
165540	T → A	12	S → T	1	0	0
165575	C → T	24	no change	1	0	0
165589	T → C	29	V → A	44	5	2
165681	A → G	59	no change	1	0	0
165717	T → G	72	W → G	8	0	0
165768	T → C	88	no change	37	3	2
165779	C → T	92	no change	28	1	2
165797	T → C	98	no change	36	1	2
165886	C → T	128	S → F	1	0	0
165892	A → G	130	H → R	44	2	2
165944	C → T	147	no change	37	5	2

Most of non-NPC EBV samples and healthy LCLs did not exhibit any nucleotide change and the sequences were similar to the B95-8 genome. Some isolates (33.3% of non-NPC and 40.0% of healthy carriers) showed 2-6 nucleotide mutations leading to 2 specific codon exchanges. These converting amino acids were identical to those of NPC majority (V29A and H130R).

Overall, amino acid conversion was revealed in 80.3% of NPC samples which is significant compared to 33.3% in non-NPC isolates (p < 0.001) but insignificant compared to the substitution rate of 40.0% in LCLs from regional healthy EBV carriers (p = 0.074). Statistical analysis also suggested that NPC isolates displayed more BARF1 sequence diversity than isolates in other EBV-related disorders (table 2). These differences were significant in the presence of 2 (p < 0.001) and 3-4 amino acid changes (p = 0.030).

**Table 2:** Proportion of NPC cases based on sequence diversity (number of amino acid conversion) when compared to other EBV-related diseases.

Number of amino acid conversion	Frequency (N) for sample of NPC and other EBV-related diseases	NPC (%)	p value
0	21	11 (52.4)	Reference
1	4	1 (25.0)	0.593
2	39	37 (94.8)	< 0.001
3 and 4	7	7 (100.0)	0.030

The presence of 3 and 4 mutations were put in one analysis to enhance statistical power.

### Phylogenetic Analysis

**Figure 1:**
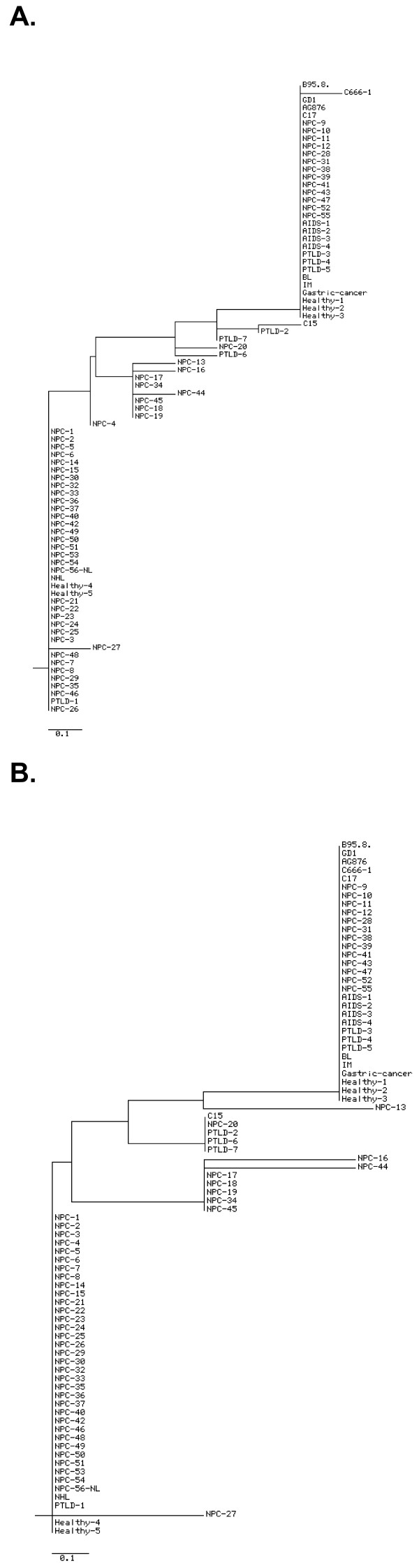
**Phylogenetic tree of individual BARF1 gene variants at the nucleotide level (A) and at the amino acid level (B)**. AIDS: Acquired immune deficiency syndrome; PTLD: post-transplant lymphoproliferative disorder; IM: infectious mononucleosis; BL: Burkitt's lymphoma; NPC-NL: NPC from The Netherlands (Dutch NPC); NHL: non-Hodgkin lymphoma. The scale reflects the evolutionary distance.

The sequences from all isolates were applied to construct a phylogenetic tree to verify the genetic difference among EBV isolates from all groups either in nucleotide or amino acid level (figure 1). The samples were characteristically clustered according to their sequence variations. There was little difference in display between the tree of nucleotide and amino acid as many nucleotide mutations consisted of silent exchanges.

At amino acid level the tree was divided into several branches with the common mutations at codon 29 and 130. The group with most of non-NPC and healthy samples in term of their similarity to the sequence of B95-8, GD1 and AG876 contained only few NPC isolates. In contrary, majority of NPC were included in other branches along with a small number of non-NPC and Indonesian healthy LCLs characterizing the occurence of more mutations and diversities. One NPC (NPC-20) and 3 PTLD isolates (PTLD-2, PTLD-6, and PTLD-7) were put in a separated branch from other isolates revealing same codon conversions (V29A and H130R) because they had less overall nucleotide mutations. Seven NPC isolates were grouped together as the fact of having 3 mutations. Two of them (NPC-16 and NPC-44) had further separation than the other 5 as they presented one more rare mutation.

In the NPC lines, C666-1 cell line had 1 silent mutation and was grouped together with the EBV genome prototypes, most of non-NPC and healthy samples, and few of NPC isolates at both the phylogenetic tree. In this group the C17 tumor line was also classified since it exhibited identical BARF1 gene sequence to B95-8. The C15 tumor line, which showed 4 nucleotide exchanges, was clustered with some PTLD isolates at nucleotide tree. At amino acid level 1 NPC sample joined this small cluster, which was separated from most of NPC samples.

### Structural analysis of BARF1 amino acid substitutions

**Figure 2:**
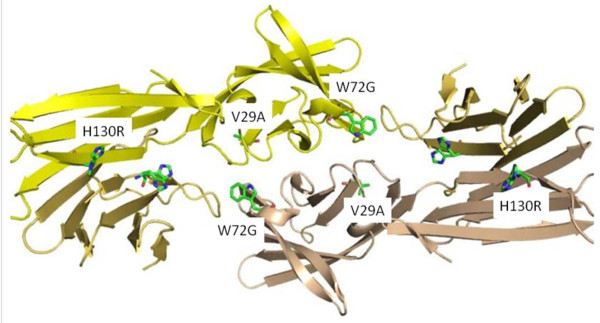
**Position of 3 typical amino acid changes in BARF1 positioned into the hexameric structure (kindly provided by dr. N.Tarbouriech)**.

Using the recently published crystal structure of hexameric B95-8 derived BARF1 we analysed possible implications of the most prevalent amino acid changes on protein secondary structure elements [17]. The three main amino acid substitutions are depicted in figure 2. The conserved V29A mutation is positioned at the start of an internal beta-sheet element and is predicted to render little change to the overall structure. The W29G mutation is providing more flexibility to a beta sheet element inside the BARF1 molecule normally stabilized by hydrophobic interactions. It is uncertain to what extent this substitutions affects the overall BARF1 structure. The H130R replacement is located towards the water-contact surface of the inner core of the BARF1 hexameric structure and may have little effect on structure, merely affecting hydrophilic water interactions. Overall the most prevalent amino acid changes in BARF1 sequence are therefore not considered to change the structural properties of BARF1.

### Correlation of BARF1 sequence variation with viral and diagnostic parameters

To determine whether BARF1 variation might associate with a particular EBV genotype, we performed a classic EBV genotype analysis for the EBNA-2 gene [13,40]. The EBV-positive JY (B95-8) DNA was used as positive control for EBV type 1 (250 bp in length) while Jijoye DNA was used as positive control for EBV type 2 (300 bp in length). EBV type 1 was detected in 55/56 (98.2%) of NPC cases and 14/15 (93.3%) of other EBV-related disorders, respectively. One NPC sample showed mixed-type infection and 1 non-NPC sample contained EBV type 2. No correlation was found between the presence of BARF1 variant to the EBV genotype, although the number of type 2 isolates was too small to allow statistical analysis.

**Figure 3:**
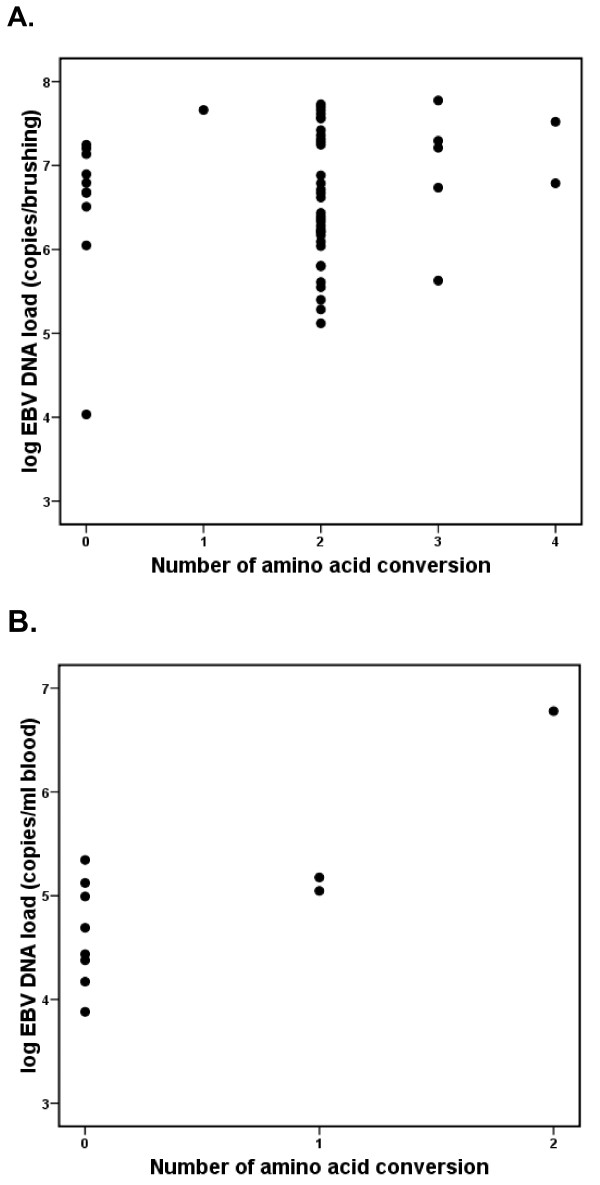
**Scatter diagram of EBV DNA load in 53 NPC brushing samples (copies/brushings) (A) and 11 non-NPC other EBV-associated isolates (copies/ml) (B) versus BARF1 amino acid conversion**. The Y axis was expressed in a log scale with cutoff value set on 2,300 copies/brushing in NPC brushing and 2,000 copies/ml in other EBV-related isolates as defined before [10]. In NPC group EBV DNA load did not relate to the presence of amino acid conversion while in other EBV-associated diseases it did (one-way ANOVA p = 0.606 and 0.014 respectively).

Severity of EBV-associated diseases can be reflected in the level of EBV DNA load in biological specimens obtained from affected patients, as demonstrated by us and others before [9,10]. Therefore we analysed EBV DNA loads in nasopharyngeal brushings and peripheral blood samples from the patients in our cohort that yielded the BARF1 isolates as shown in figure 3A and 3B. All viral DNA loads from either NPC or non-NPC samples were above the cutoff (2,300 copies/brush or 2,000 copies/ml blood). No trend or difference between the extent of BARF1 codon conversion and the level of EBV DNA load in nasopharyngeal brushings was found in the NPC samples (p = 0.606) while there was significant difference between them in blood of the non-NPC samples (p = 0.014). When considering 1 outlier in the non-NPC isolates, no significance is reached (p = 0.261).

Because BARF1 variation might have an effect on NPC tumor behaviour or EBV activity associating with the malignant process, we analysed possible correlations between BARF1 sequence variation and biological markers reflecting NPC disease progression. Most NPC samples were obtained from patients with advanced disease (tumor, nodul >2). No relation was found between the level of BARF1 variation and tumor, nodul or metastase-stage (data not shown).

**Figure 4:**
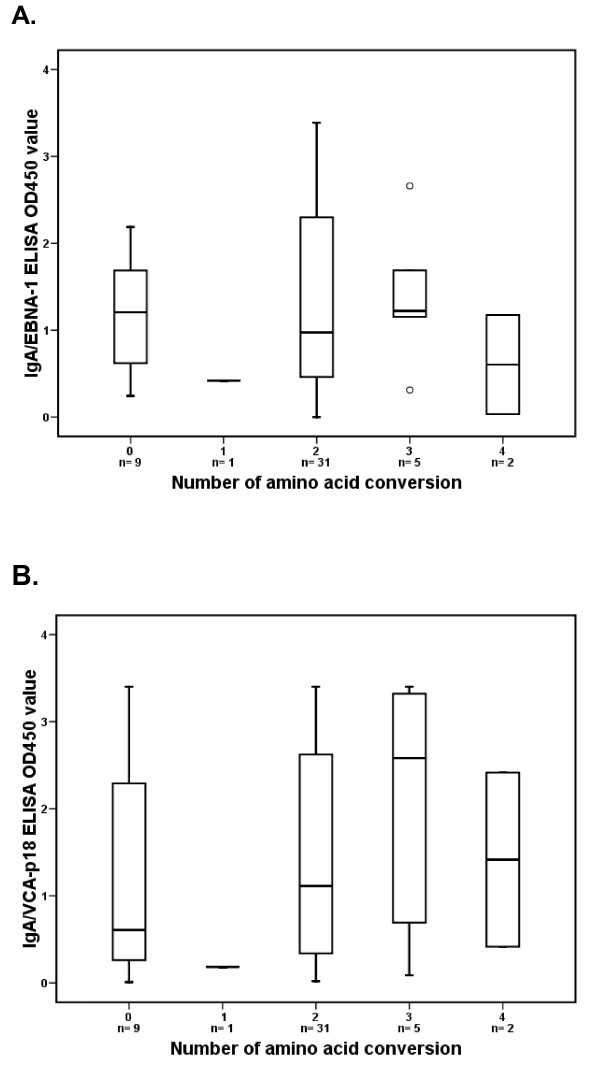
**Boxplot of seroreactivity expressed as normalized OD_450 _values of NPC sera in IgA/EBNA-1 ELISA (A) and IgA/VCA-p18 ELISA (B) versus BARF1 amino acid conversion**. The cutoff value was set on 0.3 [51]. The bar in the boxplot indicates the median value. Serology results did not relate to the presence of variant BARF1 (Mann-Whitney U test p = 0.802 and 0.382 respectively).

Increased EBV activity underlying the disease process is frequently reflected in abnormal antibody responses. Therefore we analysed whether BARF1 variation would lead to aberrant anti-EBV antibody responses in our cohort. Figure 4 shows the relation between the number of amino acid changes of BARF1 variants and serological responses of individual patients carrying this variant in the NPC group. IgA antibody levels measured by ELISA against EBV latent antigen (IgA/EBNA-1) and EBV lytic antigen (IgA/VCA-p18), shown in figure 4A and 4B. Most of NPC sera exhibited high level of IgA reactivity for both antigens. When the IgA antibody titers were compared to the presence of BARF1 amino acid conversion, no significant difference was demonstrated (p = 0.802 and 0.382 for IgA/EBNA-1 and IgA/VCA-p18, respectively). Immunoblot analysis reveals the IgG diversity underlying the overall antibody response against EBV, generally showing an elevated IgG diversity score in NPC sera. When we associated IgG diversity score to the genetic diversity of BARF1 no significant difference was proven (p = 0.058).

**Figure 5:**
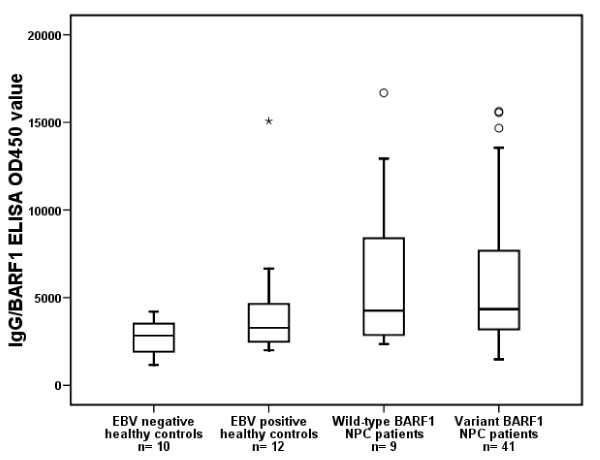
**Boxplot of seroreactivity expressed as normalized OD_450 _values of IgG/BARF1 between groups of healthy controls and NPC patients**. No difference found in anti-BARF1 response between wild-type and variant BARF1 NPC patients (Mann-Whitney U test p = 0.850).

Finally, because BARF1 mutation might have a direct influence on immune recognition and possible immune escape, we compared anti-BARF1 specific antibody responses in NPC and control patients, with known endogenous BARF1 sequence. The BARF1 used for analysis contained the most prevalent V29A and H130R. This "mutant" BARF1 protein was expressed and secreted as a hexameric molecule in human HEK293 cells and purified to homogeneity by lectin-affinity chromatography, as described in detail elsewhere (Hoebe et al., manuscript submitted). The data shown in figure 5 indicate that NPC patients and EBV seropositive controls with or without mutations in their endogenous BARF1 sequence have detectable but variable anti-BARF1 antibody responses. No statistical difference in the anti-BARF1 responses was observed between patients with B95-8 prototype or mutated NPC-related BARF1 (p = 0.850). The observed anti-BARF1 IgG reactivity was relatively low compared to anti-EBNA-1 and anti-VCA responses in the same sera (data not shown). NPC patients showed elevated but individually variable IgG reactivities to BARF1 which were different compared to healthy EBV carriers. This may be of relevance for NPC serodiagnosis.

## Discussion

BARF1 is a putative EBV-encoded oncogene, predominantly and abundantly expressed in EBV-associated carcinoma, but not in EBV-driven lymphomas [30,39,41]. To characterize genetic diversity of the EBV-encoded BARF1 oncogene, we analysed BARF1 nucleotide sequences in various EBV-positive clinical specimens. Since BARF1 is especially expressed in epithelial tumor cells, the focus was on EBV-driven NPC tumors. This study is the first to describe in detail the sequence variation of the BARF1 genotype in NPC, non-NPC EBV isolates, and healthy carriers.

A total of 11 nucleotide changes were found among NPC isolates, most of them being silent mutations. Five mutations led to amino acid changes, mostly yielding conserved mutations of V29A (valine to alanine) and H130R (histidine to arginine). Zhang et al. [42] reported variants of BARF1 gene on EBV cell lines from EBV-associated T/NK-cell lymphoma showing change of G to A at the position of 165554, change of T to C at 165589, and change of T to C at 165545. Only nucleotide mutation on 165589 (T to C) was similar to most of the sequences in our isolates. We did not find any isolate exhibiting mutation on other two nucleotides identified by that study.

Analysis of BARF1 gene in NPC and non-NPC EBV-related disorders showed a significant correlation between variant BARF1 and NPC susceptibility (p < 0.001). Interestingly, the Dutch-Caucasian NPC case (named NPC-56-NL in figure 1) also revealed a mutated BARF1 sequence. In further analysis difference in mutation frequency showed significant difference between NPC and European non-NPC group in particular the existence of 2 (p < 0.001) and 3-4 codon mutations (p = 0.030). However, no difference was demonstrated between NPC and regional EBV isolates in spontaneous LCL cultured directly from the arm of healthy Indonesian EBV carriers (p = 0.074). This indicates that mutant BARF1 EBV is prevalent in the endemic NPC population in Indonesia. Furthermore, BARF1 variants with 2 amino acid changes (V29A and H130R) most commonly detected in EBV isolates from the NPC group which indicates this to be the dominant strain in Indonesian NPC. The similarity to EBV strains found in healthy Indonesian individuals supports the view that this represents a geographic-associated distribution of EBV variants rather than a specific oncogenic strain. Moreover, the EBV-positive NPC cell line C666-1 obtained from a Chinese NPC case [43] and NPC tumor line C17 of European origin [44] were in the same phylogenetic branch with EBV genome prototype B95-8. Another NPC tumor line derived from North-African origin, C15 [44], even though demonstrating the V29A codon conversion, was also separated from most of the Indonesian NPC samples. Therefore, grouping most Indonesian NPC isolates apart from these NPC lines, as well as Chinese NPC prototype GD1, indicates that Indonesian NPC evolved independently from NPC strains from different geographic origins.

Several studies on LMP1 genetic variation argued that the genetic diversity in LMP1 gene may lead to potential immune evasion of infected host cells [45-47]. Another study showed a greater oncogenic potential of LMP1 variants with a 30 bp C-terminal deletions [24]. The overall conserved character of the BARF1 gene suggests the importance of BARF1 function for virus-driven immortalization and escape strategies in an epithelial context. This is in line with the low variability of other lytic cycle proteins, like the viral IL10 encoded in BCRF1 [48] and the major envelope protein gp350/220 encoded in BLLF1 [49]. The limited overall sequence diverged at the protein level in different EBV isolates suggests BARF1 to play an important role in epithelial persistence during natural viral infection in man.

The functional consequence of any mutation in BARF1 gene presented in this study is not known yet. Probably these will be minimal because the mutations are rather conserved. Detailed functional analysis of BARF1 protein is now underway. Our preliminary data indicate that the Indonesian variant BARF1 (V29A and H130R) is rapidly secreted from human epithelial cells, has a stable hexameric structure and binds M-CSF (CSF-1) with high affinity (Hoebe et al., unpublished data). Therefore a contribution of the observed amino acid substitutions to altered protein functions and oncogenesis is considered unlikely, but this remains to be determined in further studies.

Our ongoing studies indicate that the natural hexameric BARF1 is a protein of low immunogenicity relative to other EBV proteins and largely escapes humoral immunity. However, when present, anti-BARF1 antibody responses are formed irrespective of the sequence of the endogenous BARF1 in a particular patient. This argues against major influences in the tertiary structure imposed by the observed mutations and is in line with the conserved characteristics of the amino acid substitutions. Further studies are needed to reveal potential subtle influences of BARF1 sequence variation on its oncogenic or pathogenic properties that might predispose to NPC development.

EBV type 1 and type 2 differ in geographic distributions and among patient populations. We found that nearly 100% of our subjects contain EBV type 1, either from Asian (Indonesian) or European background. It has been recorded that EBV type 1 is predominated in Asia NPC (86.5-95.0%), while EBV type 2 is rarely detected (4.0-13.5%) [24,25,50]. Our NPC samples showed even less EBV type 2 and in a form of mixed type (1.7%). We did not find an association between EBNA-2 subtypes and BARF1 sequence variation.

Quantitative analysis of EBV DNA levels in brushing in the NPC group indicated no trend or differrence when having a BARF1 variant. In term of antibody response against EBV, all serology assays assessing the abundance or diversity of antibody responses to EBV latent or lytic antigens revealed no significant differences relating to the presence of BARF1 mutations.

## Conclusion

The present study revealed that Indonesian NPC-derived EBV strains exhibit BARF1 genetic variation relative to the B95-8 prototype which may contribute to a biological role for BARF1 in NPC development. In term of EBV genotype, viral load, and antibody reactivity to EBV there was no correlation between presence of single or multiple mutation(s) or none, suggesting no effect of BARF1 mutation of the biological markers associating with EBV carcinogenesis in NPC. The silent or conserved nature of most mutations and comparison to indirect disease markers suggests these variations are determined by the geographical prevalence and have minor biological effects on NPC development. The evolutionary conservation of BARF1 sequence and structure points to an important role for BARF1 protein in EBV biology.

## Materials and methods

### Patients and controls

Fifty-five NPC patients were recruited from Dr. Sardjito Teaching Hospital, Universitas Gadjah Mada, Yogyakarta, Indonesia. NPC diagnosis was based on pathological assessment tumor biopsy specimens based on WHO criteria, as detailed before [6,51]. All cases were in advanced stages. Approval of the local medical ethical committee was obtained. We also included 1 NPC patient identified at VU University Medical Center, Amsterdam, The Netherlands.

As controls, whole blood or tumor tissue of other EBV-related diseases (n = 15) were collected. This non-NPC group consisted of post-transplant lymphoproliferative disease (PTLD) (n = 7), acquired immune deficiency syndrome (n = 4), Burkitt's lymphoma (n = 1), non-Hodgkin lymphoma (n = 1), infectious mononucleosis (n = 1), and gastric cancer (n = 1). All non-NPC EBV-related patients were Caucasians. In order to define BARF1 variation in the general population, 5 spontaneous LCLs established from peripheral blood mononuclear cells of healthy EBV-seropositive individuals from Indonesian background, were also included. These spontaneous LCL lines were obtained by culturing fresh blood B-lymphocytes in the presence of 1 mM cyclosporine-A. NPC xenograft samples C15 and C17 were a gift from Dr. Pierre Busson (Inst. Gustave Roussy, Villejuif, France) [44]. EBV positive cell lines B95-8 and JY (EBV-1 prototype), Jijoye (EBV-2 prototype) and spontaneous LCLs were cultured in RPMI-1640 medium (Nissui Pharmaceutical Co., Tokyo, Japan) and the EBV positive NPC cell line C666-1 [43] was cultured in DMEM/Ham's F12 medium, all with penicillin (100 IU/ml), streptomycin (100 μg/ml), and 10% fetal bovine serumin a CO_2 _incubator at 37°C.

### Biological samples

Genomic DNA from NPC patients was obtained from nasopharyngeal brushings performed by experienced Ear, Nose, and Throat specialists. In all cases, the nasopharyngeal (NP) brush sample was taken prior to the biopsy in patients with suspected NPC, and both were sampled from the same site, as defined by nasendoscopy, exactly as described before [10]. In total, 56 NP brush samples were obtained from patients with subsequently biopsy-proven NPC. Genomic DNA from other non-NPC EBV-related patients were obtained either from formalin fixed paraffin-embedded tissues from archival cases or from peripheral blood [9,52].

### DNA extraction

EBV DNA was isolated from 1 ml of lysed brushings, blood or tissue samples by silica-based extraction [9,10,53]. Nucleic acids were eluted in 100 μl of which 5 μl was used in subsequent DNA amplification assays. All reagents for nucleic acid isolation were obtained from BioMerieux (Boxtel, The Netherlands).

### DNA sequencing

Primer sequences were derived from the B95-8 prototype sequence [GeneBank accession No. V01555]. We designed two pairs of primers for sequence analysis of BARF1 in a double PCR procedure. The outer sequences were BARF1-F1 (forward): 5'-TCCTCACAAACACAGAATCTG-3' (165364-165384) and BARF1-R4 (reverse): 5'-ACGAGTCGCGAGGCTATC-3' (166229-166246) amplified the full 882 bp BARF1 sequence in a first PCR step and the the inner primers BARF1-F6 (forward): 5'-GAGTGGCCTTTCAGGGGCTTC-3' (165711-165734) and BARF1-R5 (reverse): 5'-GTGGCCTTTCAGGGGCTTC-3' (165845-165866) combined with F1 and R4 were subsequently used for generating two PCR amplicons with an overlap of 155 basepairs. BARF1 amplification was performed in a final volume of 25 μl of a master mix sample solution containing 1.5 mM MgCl_2_, 200 μM dNTPs, 1 U of Taq DNA polymerase, and 10 pmol each of forward and reverse primers. The EBV B95-8 transformed LCL, JY, was used as positive control. The cycling parameters were 94°C for 5 min, 45 cycles of 94°C for 1 min, followed by 54°C for 1 min; 72°C for 1 min; and a 7 min hold at 72°C. After amplification, the PCR products were purified using QIAquick PCR Purification Kit (QIAGEN, Hilden, Germany) and 2 μl DNA was used for sequencing. PCR direct sequencing of BARF1 was carried out using BigDye Terminator version 3.1. (Applied Biosystems, Foster City, CA, USA) with 2 μl of each sample and 2 pmol of each of the corresponding primers. Each reaction was analysed on ABI PRISM 310 Genetic Analyzer (Applied Biosystems) and partly by external commercial party (Baseclear, Leiden, The Netherlands). Data were assembled and edited using Chromas Lite software (Technelysium Pty Ltd). The acquired BARF1 sequence then was aligned and compared to the combined Raji and B95-8 prototype as the wild type of EBV genome in BLAST (National Center for Biotechnology Information: NCBI; http://www.ncbi.nlm.nih.gov).

### Phylogenetic analysis

To analyse the phylogenetic relationship within the EBV sequence samples, the BARF1 sequence was aligned from B95-8, GD1 [Genbank No. AY961628], AG876 [Genbank No. NC009334], NPC lines and all BARF1 variants. The sequences were aligned and the distances between each sample were determined with the ClustalW home page. A phylogenetic tree was assembled from these matrices by the neighbor-joining method using the Phylogenetic Tree Printer (Phylodendron: http://iubio.bio.indiana.edu/treeapp/).

### EBV typing

Definition of EBV type 1 or type 2 was determined by nested-PCR [13,40]. The first PCR reaction amplified a common region of EBNA-2 followed by two separate nested reactions amplifying distinctive regions. DNA was amplified in 25 μl reactions containing 1.5 mM MgCl_2_, gelatin 0.001%, 0.3 μM of each primer, 1 U of Taq Platinum DNA polymerase (Invitrogen) and 1 μl of first reaction for nested-PCR. Cycling conditions: First reaction: 94°C 2 min, 35 cycles of 94°C 1 min, 52°C 90 sec,72°C 4 min, followed by 72°C for 10 min. Nested reaction: 94°C, 2 min, 35 cycles of 94°C 30 sec, 52°C 1 min, 72°C 2 min, followed by 72°C for 10 min. The EBV-positive JY and Jijoye were used as positive control for type 1 and type 2 PCR assays, respectively. PCR reactions were performed at least twice, in a 9800 Fast Thermal Cycler (Applied Biosystems). After PCR assay, the amplified products were subjected to electrophoresis in 2% agarose gel (Sigma), stained with ethidium bromide, and visualized under ultra violet illumination. The amplicons of EBV type 1 and type 2 were 250 bp and 300 bp in length, respectively.

### EBV DNA load quantification by quantitative real-time PCR

The EBV DNA load in brushings was determined by a quantitative LightCycler (LC) real-time PCR that targets a highly conserved 213-bp region of EBNA-1 [54]. The primers used in this assay were QP1 and QP2, and the fluorigenic internal hybridization probes were EBNA LCN and EBNA FLN (TIB MolBiol, Berlin, Germany). Real-time PCR reagents were obtained from Roche Diagnostics (Almere, The Netherlands). Ten fold dilutions of spectrophotometrically quantified plasmid DNA containing the EBNA-1 target sequence were used to create a standard curve. PCR runs were conducted in a mixture of 20 μl containing 20 pmol of each primer, 4 pmol of each probe, 2 μl of LightCycler Fast Start DNA master hybridization probe reaction mixture, 4 mM MgCl_2_, and 5 μl of plasmid DNA or clinical specimen DNA. Amplification was conducted for 45 cycles of 10 sec at 95°C, 10 sec at 55°C, and 10 sec at 72°C. Then PCR samples were cooled to 40°C.

### EBV serology

In all NPC patients and controls EBV specific IgA reactivity was quantitatively assessed by a synthetic peptide based enzyme-linked immunosorbent assay (ELISA) for immunodominant epitopes derived from EBNA-1 and VCA-p18 (BFRF3) [51]. IgG seroreactivity against EBV was assessed by immunoblotting by using the nuclear fraction of HH514.c16 cells, which were chemically induced to express the viral early and capsid antigens. Immunoblots were scored semiquantitatively from 1 (weakest) to 4 (strongest), with reference to several control samples and monoclonal antibodies analysed in parallel as described elsewhere [6].

IgG reactivity against BARF1 was studied by ELISA using sera of EBV negative and EBV positive healthy donors as well as NPC patients. The antigen for ELISA consisted of affinity chromatography-purified native hexameric NPC-derived BARF1 protein expressed in human HEK293 cells (Hoebe et al., manuscript submitted). In short, 10 μg/ml BARF1 protein was coated in 0.05 M Na_2_CO_3 _pH 9.6 overnight at 4°C. Serum dilution and incubation conditions were standardized as described before [51].

### Statistical Analysis

Chi-square test and Fischer's exact test were performed to analyse the frequency of mutation in NPC cases, non-NPC EBV-related diseases and healthy individuals. Differences between diagnostic parameters and sequence variations were tested using one-way analysis of variance (ANOVA) (EBV DNA load) and Mann-Whitney U-test (serology). Computations were done using the statistical software SPSS version 15.0.

## Competing interests

The authors declare that they have no competing interests.

## Authors' contributions

JMM and SJCS conceived the study. SHH carried out the molecular genetic studies and sequence allignment, performed statistical analysis, interpreted the data and wrote the initial draft of the manuscript. EKH purified BARF1, carried out the BARF1 antibody assay and participated in revising the manuscript. SAWMV participated in molecular work and sequence allignment. JF carried out EBNA-1 and VCA-p18 antibody assays. BH managed clinical material collection. SJCS carried out molecular work, primer design for BARF1 sequencing and sequence allignment and participated in revising the draft. JMM and AEG had primary responsibility for commenting and editing the final manuscript. SMH participated in general supervision and edited the manuscript. All authors provided comments of various drafts, participated in direction setting discussions and reviews and have read and approved the final version.
